# First episode psychosis: register-based study of comorbid psychiatric disorders and medications before and after

**DOI:** 10.1007/s00406-020-01139-6

**Published:** 2020-05-26

**Authors:** Pontus Strålin, Jerker Hetta

**Affiliations:** Department of Clinical Neuroscience, Karolinska Institute, Stockholm, Sweden

**Keywords:** Schizophrenia, Anxiety, Affective disorder, Self-harm, Personality disorder

## Abstract

Comorbid psychiatric disorders are common in first episode psychosis. We investigated comorbid disorders before, at, and after a first hospital-treated psychosis in a naturalistic nation-wide cohort (*n* = 2091) with a first psychosis hospitalization between 2007 and 2011, and at ages between 16 and 25. Swedish population registers were used to identify the cohort and to collect data on diagnoses at hospitalizations and medications. The proportions of cases with hospitalizations or medications increased year by year before and decreased in the years after the first psychosis hospitalization. In the 2 years before, 30% had hospitalizations with other psychiatric diagnoses and 60% had psychiatric medications. At the first psychosis hospitalization, 46% had other comorbid psychiatric diagnoses or self-harm. In the 2 years before or at the first psychosis hospitalization, 17% had anxiety or stress disorders at hospitalizations, 12% depressive disorders, 5.4% manic or bipolar disorders, 8.6% personality disorders, 26% substance use disorders, and 15% neurodevelopmental disorders. 8.2% had hospitalizations for self-harm. At most, around 30% of the cases were estimated not to have had any comorbid psychiatric disorders before or at the first psychosis presentation. Early comorbid affective, anxiety or personality disorders or self-harm were associated with a worse outcome, as measured by new psychiatric hospitalizations. The outcome was worst for personality disorders with 73% re-hospitalizations within 1 year and for patients with self-harm with 70% re-hospitalizations. In conclusion, most cases with a first psychosis hospitalization had clinical presentations indicating comorbid psychiatric disorders. Cases with comorbidity had a higher risk for re-hospitalizations.

## Introduction

It is well known that the clinical onset of psychotic disorders such as schizophrenia is often preceded by a period of variable length with progressive milder symptoms and decline in social and occupational function [1, 2]. In the last decades, there has been a high interest in the possibility of identifying individuals with such clinical high risk (CHR) states for psychosis, based on attenuated psychotic symptoms and functional decline [2–5]. The general aim has been to identify cases at high risk for developing severe and debilitating psychotic disorders such as schizophrenia before the emergence of fully developed disorders and to find ways of inhibiting the disease progression.

While several studies have shown that a majority of cases that have developed a psychotic disorder have had CHR symptoms before transition into psychosis [5–7], retrospective studies in catchment areas with access to CHR services indicated that only small parts of the first episode psychosis (FEP) cases were identified by the CHR services before FEP [8, 9]. There is a need to develop additional ways to identify cases at high risk of developing psychotic disorders.

Many studies have found that it is common with other types of mental symptoms and disorders before the onset of a first episode psychosis (FEP): depression and anxiety disorders are common before the onset of psychosis [6, 10–13]. The risk of developing a psychotic disorder after manic episodes is significantly increased, but the proportion of cased with FEP with previous bipolar episodes is anyhow low [9, 14]. Increased risks for psychotic disorders have also been found for individuals with neurodevelopmental disorders (NDDs), including autism, ADHD and intellectual disability [9, 15–17], for individuals with substance use disorders (SUD) [13, 18, 19], and for individuals with personality disorders. A large Danish population study found that around 10% of the cases with a diagnosis of a personality disorder later were diagnosed with a psychotic disorder [13].

A complication in the quest for early identification of emerging psychotic disorders is that many other mental disorders may involve psychotic or psychosis-like symptoms, such as affective disorders [20], personality disorders [21], PTSD [22], autism [23], and SUD [19].

With current diagnostic tools, it is not always possible in young patients with recent onset of symptoms to differentiate between an emerging psychotic disorder with other concomitant mental problems, and psychotic symptoms associated with other emerging mental disorders. Cases with emerging psychotic episodes in conjunction with other mental problems may turn out to develop schizophrenia or sometimes other mental disorders [13, 24].

From the findings sketched above, we believe that there is a need for a better understanding of the temporal relations between FEP and comorbid disorders. This may contribute to the development of methods for early detection of FEP and of cases at high risk for chronic psychotic disorders such as schizophrenia, and to development of differentiated treatment algorithms based not only on psychotic symptoms, but also on the comorbidity context of the psychotic disorders.

In the current study, we wanted to investigate to what extent cases with a first episode of hospital-treated psychosis had had medications and hospitalizations for different other mental disorders before and to what extent there were different comorbid diagnoses in conjunction with the first psychosis hospitalization. We also wanted to investigate to what extent the different comorbid disorders remained in the years after.

The study focuses on early comorbid affective disorders, anxiety and stress disorders, personality disorders, and early self-harm in relation to the first episode hospital-treated psychosis. We hypothesized that a majority of cases had comorbid disorders before or concomitant with the FEP and that the comorbid diagnosis in many cases would remain after the first episode of psychosis.

We used a Swedish national cohort based on national registers to trace hospitalizations and pharmacological treatments for psychiatric disorders before and after a first episode hospital-treated psychosis (ICD10 F20-29).

## Methods

### Data sources for the study

The unique personal identity number assigned to each permanent resident in Sweden was used to link data from different registers.

The In-patient Care Diagnoses Database includes all individuals in Sweden admitted to psychiatric or general hospitals [25, 26]. It includes dates for admission and discharge and ICD-10 diagnoses for every inpatient care episode in Sweden since 1987.

Hospitalizations for psychosis were identified by ICD-10 F2 diagnoses. Substance use disorders were identified by ICD-10 F1 diagnoses at hospitalizations. Hospitalizations for depression were identified by F32–F33 diagnoses, manic or bipolar disorder by F30–F31 diagnoses, anxiety and stress disorders by F40–F43 diagnoses, and personality disorders by F60. Neurodevelopmental disorders were identified by ICD-10 codes F7 (intellectual disability), F84 (autism) or F90 (ADHD), or for ADHD also with dispensations of psychostimulant medications (ATC code N06B).

Suicide attempts and other serious self-harm were identified by ICD10 codes X60–X84 at any type of hospitalization in the specified years.

The Swedish Prescribed Drug Database comprises information on all dispensations of prescribed medicines in Sweden, including the Anatomical Therapeutic Chemical code (ATC) of the dispensed substances, amounts, formulation and date of prescribing and dispensing since July 2005 [27]. However, it does not cover drugs administered at hospitals.

For analyses of antipsychotics, ATC code N05A was used, with the exceptions of N05AN (Lithium) used for bipolar disorder, and N05AA02 (levomepromazine), N05AD03 (melperone), and N05AF03 (chlorprothixene), seldom used for psychotic symptoms. For analyses of antidepressants, the ATC code N06A was used for sedatives N05AA02, N05BA, N05BB, N05C, and R06AD (including benzodiazepines, most non-benzodiazepine sedatives and sleeping pills); for antiepileptics N03A, for psychostimulants N06B and for medications against alcoholism N07BB.

Amounts of dispensed medications for each dispensation are reported in the register (and used in the study) as “defined daily doses” (DDD) according to the WHO ATC/DDD Index [28]. Definitions of one DDD for some common antipsychotic, antidepressant and sedative medications are: clozapine 300 mg, olanzapine 10 mg, risperidone 5 mg (2.7 mg for depot), aripiprazole 15 mg, quetiapine 400 mg, paliperidone 6 mg (2.5 for depot), sertraline 50 mg, mirtazapine 30 mg, clomipramine 100 mg, diazepam 10 mg, oxazepam 50 mg, alimemazine 30 mg and promethazine 25 mg.

Amounts of antipsychotic, antidepressive or sedative medications in the year before and the year after the first psychosis hospitalization were calculated by summation of the numbers of DDD from all dispensations of the type of medication in 1 year.

Deceased cases were identified by the Causes of Death Database, which comprises information on all deaths of Swedish residents [29].

### Study design

The study cohort was selected from the In-patient Care Diagnoses Database based on data on all hospitalizations from 1987 for the Swedish population. All individuals with a first occurrence of a hospitalization for psychosis in Sweden (as defined by the International Classification of Disease, ICD-10, F20-29) with admission after 1 July 2007 and discharge before 31 December 2011 and between age 16 and 25 at first admission were selected. 2133 cases were identified by these criteria. 42 cases had deceased before the end of 2 years after the first discharge from psychosis hospitalization. The 2091 cases alive 2 years after the discharge from the first hospitalization for psychosis were included in the cohort.

Data on hospitalizations for the cohort were collected from up to 5 years before until up to 5 years after the first psychosis hospitalizations for further analyses as described below.

Episodes of inpatient care were combined if the time between episodes in the National Patient Register was 7 days or less.

The full cohort was included in all analyses of the period from 2 years before until 2 years after the first psychosis hospitalization. For analyses of hospitalizations and dispensations of medications in the earlier or later years in relation to the index hospitalization, the number of cases included were reduced due to lack of data or death of cases. For analyses of the fifth year before the index hospitalization, data were available for 757 cases and for the fifth year after 560 cases.

### Statistical analysis

Individuals identified in several groups of diagnostic or pharmaceutical categories were included in all the identified categories.

Significance levels for relative risks were calculated based on Wald statistics. 95% Confidence intervals for proportions of cases were calculated with the exact method for binomial distributions.

Significance levels for the differences in the median amounts of antipsychotics were calculated with the Wilcoxon method due to non-normal distribution of the amounts.

Descriptive statistics and relative risk calculations were made with the R software [30].

The study was approved by the Regional Ethical Review Board in Stockholm, Sweden (ref 2014/481-31/4).

## Results

### Demographics and distribution of psychosis diagnoses at the first psychosis hospitalization

The median age at the intake to the first psychosis hospitalization was 21.2 years. 64% of the cases were male. Psychosis diagnoses were distributed as follows at the first psychosis hospitalization: schizophrenia (F20) 11%, delusional disorder (F22) 5%, acute and transient psychotic disorders (F23) 36%, schizoaffective disorder (F25) 4%, other nonorganic psychotic disorders (F28) 1.2% and unspecified nonorganic psychosis (F29) 42%. Cases were hospitalized for a median of 18 days at the first psychosis hospitalization with 25% and 75% quantiles of 7 and 41 days. Of the cases with unspecified psychosis at the index hospitalization, and with any subsequent new hospitalizations within the follow-up time, 22% later received a schizophrenia or a schizoaffective diagnosis at a hospitalization.

42 cases had deceased before the end of 2 years after the index hospitalization and were excluded from the study population. 32 of the deceased cases were suicides. Of the suicide cases, 13 cases (42%) had had psychiatric hospitalizations in the 2 years before the index hospitalizaiton. The most common diagnoses in the 2 years before or comorbid at the index hospitalization were SUD (ICD10 F1) in 9 cases (28%) and affective diagnoses (F3) in 9 cases. 8 cases (25%) had dispensed antidepressive medications and eight cases antipsychotic medications in the 2 years before. Analyses of mortality in the cohort have previously been published [31].

### Proportions of cases with psychiatric hospitalizations in the years before, or concomitant psychiatric diagnoses at the first psychosis hospitalization

**Fig. 1:**
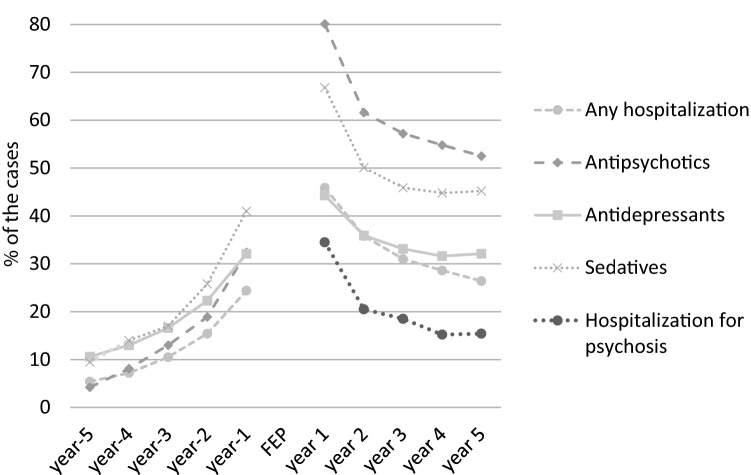
Proportion of cases with hospitalizations or medications from 5 years before the first psychosis hospitalization until 5 years after. The full cohort was used for the calculations from 2 years before until 2 years after. Only cases with full years of observation were included in the analyses of earlier or later years, see under methods. “FEP” indicate the first psychosis hospitalization

The proportions of cases with psychiatric hospitalizations and medications increased from low levels year by year in the 5 years before the first hospitalization for a psychotic disorder (Fig. 1). In the year before the index hospitalization, 24% of the cases had any hospitalization, and in the 2 years before, the proportion was 30% (Fig. 1, Table 1). 46% of the cases had at least one comorbid psychiatric diagnosis or a diagnosis for self-harm at the first psychosis hospitalization (Table 1). Together, 56% of the cases in the cohort had at least one other diagnosis (or selfharm) at hospitalizations in the 2 years before or at the first psychosis hospitalization.

**Table 1:** Overview of comorbid disorders at hospitalizations and medications before, at and after the first psychosis hospitalization (indicated as FEP in the table)

	2 years before or at FEP	2 years before FEP	at FEP	1 year after FEP
Hospitalizations for				
SUD	551 (26%)	307 (15%)	391 (19%)	305 (15%)
Manic/bipolar	112 (5.4%)	60 (2.9%)	71 (3.4%)	83 (4%)
Depression	257 (12%)	157 (7.5%)	126 (6%)	131 (6.3%)
Anxiety/stress	369 (18%)	209 (10%)	221 (11%)	184 (8.8%)
Personality disorder	179 (8.6%)	106 (5.1%)	135 (6.5%)	139 (6.6%)
NDD	316 (15%)	210 (10%)	223 (11%)	258 (12%)
Self-harm	172 (8.2%)	116 (5.5%)	75 (3.6%)	118 (5.6%)
New psychosis hospitalizations				722 (35%)
Any comorbid diagnosis at a hospitalization	1172 (56%)	629 (30%)	960 (46%)	713 (34%)
Medications				
Antipsychotics		785 (37%)		1674 (80%)
Antidepressants		736 (35%)		927 (44%)
Sedatives		987 (47%)		1396 (67%)
Any psychiatric medication:		1254 (60%)		1869 (89%)
Any hospitalization or medication		1316 (63%)		1929 (92%)
Any medication or comorbid diagnosis at hospitalizations	1579 (75%)			

Proportions of the cohort with disorders, self-harm and medications are presented. Cases may be included in several groups of diagnoses and medications and both in the columns for “2 years before FEP” and for “at FEP”. Data on medications during hospitalizations were not available. *n* = 2091 for the cohort

12% of the cases had depressive disorders (F32–F33) at hospitalizations in the 2 years before or at the first psychosis hospitalization (Table 1). 21% of the cases with depressive disorders in the 2 years before had any depression with psychotic symptoms (F32.3/F33.3).

5.4% of the cases had manic or bipolar disorders (F30/F31) at hospitalizations in the 2 years before or at the first psychosis hospitalization.

18% of the cases had anxiety or stress-related disorders (F40–F43) at hospitalizations in the 2 years before or at the first psychosis hospitalization. 20% of them had mixed anxiety and depressive disorder (F41.2), 35% had unspecified anxiety (F41.9), 14% had OCD (F42), 16% had acute stress reactions (F43.0) and 11% had PTSD (F43.1).

8.6% of the cases had personality disorders (F60) at hospitalizations in the 2 years before or at the first psychosis hospitalization. The most common type was emotionally unstable personality disorder (ICD10 F60.3), diagnosed in 53%, followed by unspecified personality disorder (ICD10 F60.9) in 44%.

8.2% of the cases had suicide attempts or serious self-harm at any type of clinic, including emergency units, at hospitalizations in the 2 years before or at the first psychosis hospitalization. Intoxications (ICD10 X6) were the most common form of self-harm, occurring in 87% of the cases, followed by cutting (ICD10 X78) in 15% of the cases. 38% of the cases had a diagnosis of personality disorder at hospitalizations in the same period.

26% of the cases had a substance use disorders (SUD, ICD10 F1) at hospitalizations in the 2 years before or at the first psychosis hospitalization. 15% of the cases had a neurodevelopmental disorder (NDD, either intellectual disability, ICD10 F7, autism, F84, or ADHD, F90) identified as diagnoses at hospitalizations in the 2 years before or at the first psychosis hospitalization, or for ADHD also by dispensing of psychostimulants the 2 years before (Table 1). Analyses of the NDDs [15] and SUD [32] in the cohort have previously been published, and will thus not be further explored in the present paper.

Public databases at the Swedish national board of health and welfare [25], report that hospitalizations in the general population in the ages between 20 and 24 years old in 2009 were as follows: 0.18% of the population were hospitalized for affective disorders (F3) in 1 year, 0.21% for anxiety disorders (F4), 0.076% for personality disorders and 0.24% for self-harm.

Cases with a schizofrenia diagnosis (F20) at the index hospitalization did not have significantly different probabilities for hospitalizations for other psychiatric disorders in the 2 years before compared to cases with other psychosis diagnoses.

### Proportions of cases with psychiatric medications in the years before the first psychosis hospitalization

60% of the cases had any dispensation of psychiatric medications in the 2 years before the first psychosis hospitalization. 47% had dispensations of sedatives, including benzodiazepines, non-benzodiazepine sedatives and sleeping pills, 37% of antidepressive medication (54% had dispensations of sedatives or antidepressive medications, Fig. 1), 35% of antipsychotic medication, 12% of antiepileptics, 6.7% of Psychostimulants, 2.3% of lithium and 2.9% of medications against alcoholism. Analyses on psychostimulants in the cohort have previously been published [15].

58% of the cases with antipsychotic medication in the 2 years before the first psychosis hospitalization had some inpatient care for other mental disorders in the same period. Of the cases with both antipsychotics and any hospitalizations before the index hospitalization, 38% had inpatient care for SUD, of which 41% had SUD with psychotic symptoms, 12% had inpatient care for manic episodes or bipolar disorder, 25% for depressive disorders and 36% for anxiety- and stress-related disorders. 55% of the cases with antipsychotic medication in the 2 years before had at least one other diagnosis at the first psychosis hospitalization and 76% had other concomitant diagnoses either in the 2 years before or at the first psychosis hospitalization.

We analyzed the temporal relations between other diagnoses and antipsychotic medication before the first psychosis hospitalization by estimating the proportion of cases with other medications or any hospitalization before the first dispensation of neuroleptic medication. There were 1684 cases with data on dispensations of medications for at least 3 years before the first psychosis hospitalization. In that group, 84% of the cases with antipsychotics for the first time in the 2 years before had had psychiatric hospitalizations or dispensations of other psychiatric medications before the first dispensation of antipsychotics. 16% of the cases with antipsychotic medication in the 2 years before the first psychosis hospitalization, equaling 5.6% of the cohort, were thus estimated to have had antipsychotic medication as the first traceable psychiatric intervention. We consider this as a proxy for possible psychotic symptoms as a first clinical presentation.

51% of the cases with dispensations of antidepressant medication in the 2 years before the first psychosis hospitalization had hospitalizations for mental disorders other than psychotic disorders in the same period.

In the full cohort, 45% of the cases had indications of a depressive disorders, or an anxiety- or stress-related disorders before or concomitantly with the first hospital-treated psychosis, as reflected in diagnoses at hospitalizations or prescriptions of antidepressive medication, using antidepressive medications as a proxy for a depressive- or anxiety-related disorder.

37% of the cases in the cohort had no psychiatric hospitalizations, hospitalizations for self-harm, or dispensations of any psychiatric medications in the 2 years before the index hospitalization. These cases were more likely to be diagnosed with a acute and transient psychotic disorders (ICD10 F23) at the first psychosis hospitalizations (OR 1.7, *p* value < 0.001).

25% of the cases in the cohort did not have any psychiatric medication in the 2 years before or any comorbid psychiatric disorder at hospitalizations in the 2 years before or at the first psychosis hospitalization.

We estimate the proportion of cases with a psychotic presentation without comorbid psychiatric disorders at the first contact with psychiatry to be at most 31% of the cases in the cohort, based on the 25% without indications of comorbidity or previous medications at the index hospitalization, plus 5.6% with dispensing of antipsychotic medication before, as the first traceable psychiatric event (see above).

### Gender distribution of comorbid disorders and medications

We analyzed the gender distribution of the other mental disorders at hospitalizations in the 2 years before or at the first psychosis hospitalization and of the psychiatric medications in the 2 years before. There were significant differences in the distribution of disorders and medications between genders (Table 2). Affective disorders, anxiety and stress disorders, personality disorders and self-harm were more frequent in women. Treatments with antidepressive, sedative and antipsychotic medications before the first psychosis hospitalization were also more common in women.

**Table 2:** Gender distribution of comorbid psychiatric disorders in the 2 years before or at the first psychosis hospitalization and psychiatric medications in the 2 years before

	2 years before or at FEP	Men	Women	RR	*p* value
Cohort	2091	1336	755		
Manic/bipolar disorder	112	55 (4%)	57 (8%)	1.8	0.001
Depressive disorder	257	126 (9%)	131 (17%)	1.8	< 0.0001
Anxiety or stress disorder	369	176 (13%)	193 (26%)	1.9	< 0.0001
Personality disorder	179	66 (5%)	113 (15%)	3	< 0.0001
Self-harm	172	67 (5%)	105 (14%)	2.8	< 0.0001
Antidepressive medication	785	431 (32%)	354 (47%)	1.5	< 0.0001
Antipsychotic medication	736	438 (33%)	298 (39%)	1.2	0.0019
Sedative medication	987	548 (41%)	439 (58%)	1.4	< 0.0001

Relative risks (RR) were calculated as the risk for the disorder for women in relation to the risk for men

### Medications and hospitalizations in the years after the index hospitalization

47% of the cases had any new psychiatric hospitalization in the first year after the first psychosis hospitalization. 35% had new hospitalizations with psychosis diagnoses (ICD10 F20–F29), and 34% had new hospitalizations with other psychiatric disorders (Table 1). Of the cases with a schizophrenia diagnosis (ICD10 F20) at the index hospitalization, 52% had any new hospitalizations, 46% had new hospitalizations with psychosis diagnoses corresponding to a significantly higher risk compared to cases with other psychosis diagnoses (RR = 1.5, *p* < 0.001) and 29% had new hospitalizations with other psychiatric disorders.

The distributions of diagnoses at new hospitalizations and of medications in the first year after the index hospitalization are presented in Table 1. In the full cohort, 47% of the cases had indications of a depressive disorder, or an anxiety- or stress-related disorder as reflected in diagnoses at hospitalizations or dispensations of antidepressive medication in the first year after the first hospital-treated psychosis, and 55% in the 2 years after.

The proportion of cases with hospitalizations decreased year by year after the index hospitalization (Fig. 1). Similar patterns of decreasing proportions in the first years were noted for the psychiatric medications analyzed (Fig. 1).

8% of the cases did not have any dispensation of psychiatric medication or any new hospitalization in the first year after discharge from the first psychosis hospitalization.

The median amounts of antipsychotic medication dispensed among cases with any dispensation during the year before the index hospitalization was 97 DDD with 25% and 75% quantiles at 28 and 250 DDD. The amounts increased significantly in the first year after the index hospitalization to a median level of 216 DDD with 25% and 75% quantiles at 93 and 420 DDD. For antidepressives, the amounts dispensed increased less between the year before and after the index hospitalization. The median level the year before was 228 DDD with 25% and 75% quantiles at 100 and 471 DDD and the year after 298 DDD with 25% and 75% quantiles at 129 and 500 DDD.

### Outcome for cases with concomitant psychiatric disorders (including self-harm) preceding or at the first psychosis hospitalization

**Fig. 2:**
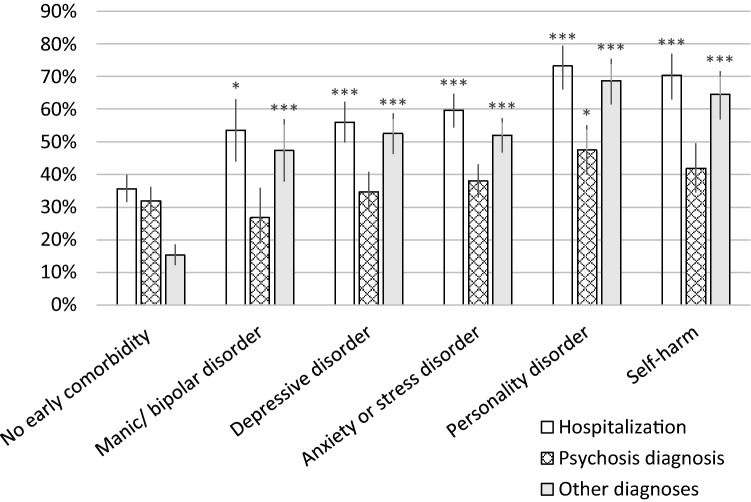
Outcome in terms of new hospitalizations with any psychiatric diagnoses, with psychosis diagnoses or with other psychiatric diagnoses in the first year after the index hospitalization. Proportions of cases with new hospitalizations are presented for groups with different comorbid psychiatric disorders at hospitalizations in the 2 years preceding or co-occurring with the index hospitalization. The “No early comorbidity” group includes the 25% of the cases with no psychiatric hospitalizations or medications in the 2 years preceding, or comorbid psychiatric disorders at the index hospitalization. 95% confidence intervals for the proportions are indicated as error bars. Significance levels for the differences in proportions between groups with early comorbid disorders and the group with no early comorbidity are presented as * for a *p* value < 0.05, **for a *p* value < 0.01, and ***for *p* value of < 0.001

Figure 2 presents the risks for new hospitalizations in the first year after discharge from the index hospitalization for groups with different earlier diagnoses: three types of hospitalizations are presented. First, the risk for any new psychiatric hospitalizations, then for new hospitalizations for psychotic disorders (F20–F29) and last the risk for new hospitalizations for other psychiatric diagnoses.

The studied groups with comorbid mental disorders before or at the index hospitalization had significantly higher risks for new psychiatric hospitalizations and for new hospitalizations with psychiatric diagnoses other than psychosis in the first year after (Fig. 2). The highest risks for any new hospitalizations were noted for cases with diagnoses of personality disorders or self-harm (Fig. 2). Cases with personality disorders before or at the first psychosis hospitalization also had a modest increase in risk of a new psychosis hospitalization in the first year (Fig. 2).

There were no significant differences in the proportions of cases that were given schizophrenia diagnoses up to 2 years after the index hospitalization between the group without early comorbidity and the groups with early comorbid affective, anxiety or personality disorders or with self-harm. For the full cohort, the proportion was 18%. For schizoaffective disorder, the proportion in the full cohort was 7%, and significantly higher (*p* values < 0.05 with Pearson's Chi-squared test) in all the groups with comorbid diagnoses. The highest proportion was noted for cases with early comorbid manic or bipolar disorder with 33% of the cases, followed by personality disorder with 18%.

Significantly, larger proportions of females had new hospitalizations for any psychiatric diagnosis and for other diagnoses than psychosis the first year after the index hospitalization. 52% of females compared to 44% of male cases had any new hospitalization (*p* value of 0.006 with Pearson's Chi-squared test). Gender did not influence the risk for a new psychosis hospitalization, the proportion for men was 35% and for women 34%.

Female cases were significantly more likely to dispense antipsychotic medication. 78% of the men had any dispensing year 1, and 83% of the women (*p* value of 0.006 with Pearson's Chi-squared test).

In a second analysis, the outcome in the cohort in terms of new hospitalizations for the different diagnoses in the first year after the index hospitalization was studied (Table 3). Relative risks were calculated for a new hospitalization for a disorder (or self-harm) in relation to a previous hospitalization for the same disorder before or at the first psychosis hospitalization.

**Table 3:** The table presents relative risks for new hospitalizations for the different disorders after the first psychosis hospitalization

Diagnostic category	RR	RR 95%CI	*p* value
Manic/bipolar disorder	10.1	6.7–15.1	< 0.0001
Depressive disorder	4.7	3.4–6.5	< 0.0001
Anxiety or stress disorder	4.6	3.5–5.9	< 0.0001
Personality disorder	17.5	12.9–23.6	< 0.0001
Self-harm	7.4	5.3–10.3	< 0.0001

Relative risks were calculated for new hospitalizations for disorders in the first year after discharge from the index hospitalization in relation to having had or not having had hospitalizations for the same disorder in the 2 years before or at the index hospitalization. 95% confidence intervals and *p* values for the relative risk values are presented

A general pattern observed was that a previous hospitalization for any of the studied disorders was a strong predictor for a new hospitalization for the same disorder after the first psychosis hospitalization (Table 3). The strongest association was noted for personality disorders.

## Discussion

In the study, we found that a majority of the cases in a naturalistic cohort of first episode hospital-treated psychosis had indications of comorbid mental disorders before or concomitant with the first hospital-treated psychosis. Many cases may have had psychotic symptoms for some time before the first psychosis hospitalization, as indicated by dispensations of antipsychotic medication and by other diagnoses with secondary psychotic symptoms in the years before. At the same time, at most around a third of the cases had a psychotic presentation without indications of comorbid disorders at the first traceable psychiatric contact.

We found a variety of earlier or concomitant comorbid disorders in the cases including substance use disorders, neurodevelopmental disorders, affective disorders, anxiety disorders and personality disorders. One approach to understanding the presentations with comorbid disorders preceding or co-occurring with the onset of a psychotic disorder is to consider the comorbid disorders as types of prodromal states or high-risk states for transition into psychosis. Rates of transition into psychosis have previously been calculated for individuals with non-psychotic mental disorders [10]. Future research on possible markers for psychosis risk within the groups of hospital-treated affective disorders, anxiety disorders or personality disorders, with self-harm, or with other psychiatric diagnoses at risk may prove fruitful [3, 33].

Affective disorders, anxiety disorders or personality disorders before or in conjunction with the onset of psychosis, tended to remain with many cases also after the first psychotic episode. Likewise, the risk for self-harm after was much higher for cases with self-harm before or at the first psychosis hospitalization. This finding suggests, in line with other studies [13], that there may be a variety of risk states for transition into psychosis and that the different risk states preceding the psychosis will influence the clinical presentations and outcomes after the transitions to psychosis.

The high risks for new hospitalizations for the comorbid disorders in the year after the index hospitalization indicate that for some of the cases, the personality disorders, affective disorders or anxiety disorders present before, or concomitantly with the first psychosis may be the more chronic and lasting. Young patients with emerging symptoms of different types, including emerging psychotic symptoms, have recently been suggested to have pluripotent “at risk states”, carrying a risk for development of different persistent mental disorders, including psychotic disorders, depressive disorder bipolar disorder, personality disorders and SUD [24]. The results in the current study indicate that many young cases with fully developed FEP may have an increased risk for development of a few different persisting psychiatric disorders, including persistent psychotic disorders, depending on the case history and the set of symptoms before and at the FEP.

The risk for new psychosis hospitalizations was not much influenced by the studied comorbid disorders. It did not differ significantly between the groups with and without early comorbid disorders, except for a slightly higher risk for cases with diagnoses of personality disorders. Cases without comorbid disorders before or at the first psychosis hospitalization had a lower risk for any new hospitalizations, indicating a more favourable overall outcome.

These observations may have implications for the management and treatment strategies of first episode psychosis [15]. The clinical presentations with different sets of comorbid disorders need to be properly investigated and treatment strategies need to be differentiated depending on the comorbidity context of the FEP.

Affective disorders, anxiety disorders, personality disorders or self-harm before or at the first psychosis hospitalization were more common in women, which may partly explain differences in presentations and course of psychotic disorders between men and women [34, 35].

The proportion of cases with psychiatric hospitalizations or medications, increased year by year before the index hospitalization, and declined year by year after. This indicates a progression of the different types of symptoms before the transition into a psychotic state in many cases, and a degree of recovery and a general reduction in symptom severity, including psychotic symptoms, on a group level in the years after. Declining proportions of cases with dispensations of antipsychotic medication were also observed in the years after. The findings suggests a rather good recovery in many of the cases after a first episode psychosis [36, 37].

Antipsychotic medication before the first psychosis hospitalization was not uncommon. Some of the cases with antipsychotic medication before likely were medicated for (milder) psychotic symptoms preceding the first psychosis hospitalization. At the same time, most cases with antipsychotic medication in the years before the index hospitalization had psychiatric hospitalizations or medications for other mental disorders before the first dispensation, and many had hospitalizations with other psychiatric disorders in the same period as the dispensations. The amounts of antipsychotics dispensed before were mostly low. Since neuroleptics are often used in low doses for other types of mental problems than psychosis, it is likely that, in many cases, they were prescribed for symptoms related to affective disorders, anxiety- and stress-related disorders, and SUD. Patients with autism are sometimes treated with antipsychotics for irritability [38]. Cases in the cohort with a diagnosis of autism have previously been reported to have a higher probability of antipsychotic treatment before the first psychosis hospitalization [15].

The study has several limitations. The study cohort only included cases with a psychosis diagnosis with ICD 10 codes F20–F29 at hospitalizations. Cases with psychotic states in conjunction with other disorders coded only by the other disorders, such as affective disorders with psychotic symptoms (e.g. severe depression with psychotic symptoms, F32.3) or psychotic disorder due to substance use(F1X.5 or F1X.7) were not included. If these diagnostic categories would have been included, the estimations of proportions of cases with comorbidity would have been higher. Cases with milder psychotic disorders, not treated in hospitals were not detected and not included in the cohort.

A larger proportion of cases in the cohort received a diagnosis of unspecified psychotic disorder (F29) at the first psychosis hospitalization compared to proportions generally reported in FEP studies [39, 40]. The high proportion of unspecified psychosis in this national cohort may reflect a hesitation from clinicians to use a diagnosis of schizophrenia in cases with recent onset of psychosis, and a less rigorous diagnostic procedure in clinical praxis compared to in research projects. There may also be cultural differences in the diagnostic procedures with for example, a longer delay in Sweden before a diagnosis of schizophrenia is set, compared to some other countries.

Several disorders were likely underestimated since only diagnoses at hospitalizations or medications in the 2 years before or at the index hospitalization were counted. Unsurprisingly, a considerably larger part of the cohort had antidepressive medications before or after the index hospitalization compared to cases with corresponding diagnoses at hospitalizations, indicating a larger proportion with disorders in the broader spectrum of depressive or anxiety disorders. ADHD was identified by both diagnoses at hospitalizations and dispensings of psychostimulants before the index hospitalization, but only in the 2 years before. Cases with only outpatient care for psychiatric disorders, and no medications, before the first psychosis hospitalization were not identified. Neither cases with hospitalizations or medications only earlier than 2 years before. Cases with any hospitalizations before the first psychosis hospitalization likely represent groups with worse symptoms and disorders, which likely have a higher risk for transition into psychotic disorders.

The validity of diagnoses in the national registers may not correspond to research standards: The general pattern was likely underestimations of cases meeting formal criteria for diagnoses. Some cases may also have been given diagnoses, for example of personality disorder, without meeting formal criteria due to a lack of structured diagnostic procedures in clinical praxis compared to if research procedures would have been used [13, 41]. It is also likely that some cases, seeking help for other symptoms and problems, also had psychotic symptoms that were not detected and diagnosed in earlier clinical contacts.

Only young cases were included in the cohort. Cases with higher age at the FEP may have different distributions of comorbid disorders and different outcomes. Since women have a higher median age at FEP, a larger part of women with FEP may have a different profile of comorbid disorders and outcomes compared to the current cohort.

In conclusion, it is clear from the current study that cases with first episode psychosis in a majority of cases also have indications of other comorbid psychiatric disorders. The clinical presentations with comorbid affective disorders, anxiety disorders or personality disorders have an overall less favorable outcome. We have previously described comorbidity with neurodevelopmental disorders [15] and with SUD [32] and how it influences outcome. The heterogeneous outcomes of cases with the studied comorbid disorders suggest that multiple trajectories of clinical development may occur, where some cases may develop dominant psychotic disorders such as schizophrenia or schizoaffective disorder, while others may develop other dominant psychiatric disorders, and some recover to various extents.

More research is needed to better understand the nature of the disorders with clinical presentations of both psychotic and other comorbid disorders, and to find differentiated treatment strategies.
